# Healthcare-associated infections in an intensive care unit: a descriptive study, Mato Grosso state, Brazil, 2018-2021

**DOI:** 10.1590/S2237-96222026v35e20250405.en

**Published:** 2026-03-30

**Authors:** Mayane Emanuelle Oliveira Fonseca, Francisco Kennedy Scofoni Faleiros Azevedo, Ana Luiza Rodrigues Silva, Marco Andrey Pepato, Wagner Izidoro de Brito, Eduarda Pavan, Cor Jesus Fernandes Fontes, Ruberlei Godinho de Oliveira

**Affiliations:** 1 Universidade Federal de Mato Grosso, Faculdade de Medicina, Cuiabá, MT, Brazil; 2 Hospital Universitário Júlio Müller, Mestrado Profissional em Ciências Aplicadas à Atenção Hospitalar, Cuiabá, MT, Brasil; 3 Secretaria de Estado de Saúde de Mato Grosso, Cuiabá, MT, Brasil

**Keywords:** Intensive Care Units, Hospital Infection Control Services, Drug Resistance, Microbial, COVID-19, Retrospective Studies, Unidades de Cuidados Intensivos, Servicios de Control de Infecciones Hospitalarias, Farmacorresistencia Microbiana, COVID-19, Estudios Retrospectivos

## Abstract

**Objective:**

To analyze the demographic and microbiological characteristics and clinical outcomes of people with healthcare-associated infections (HAI) admitted to an intensive care unit (ICU) in Mato Grosso state, Brazil, between 2018 and 2021, comparing the pre-COVID-19 and the COVID-19 pandemic periods.

**Methods:**

This is a descriptive and longitudinal study with secondary data on people diagnosed with HAI. HAI, isolated microorganisms, sensitivity profile, comorbidities, clinical outcomes and sociodemographic data were analyzed.

**Results:**

Between 2018 and 2021, 384 people were admitted to the ICU. There were 218 HAI episodes in 179 of them. In the pre-pandemic period (2018-2019), 95 episodes were recorded in 73 people, predominantly associated with: mechanical ventilation (48.4%), male sex (65.8%), the 40-64 age group (43.8%), those of mixed race (80.8%) and hypertensive (45.2%), with 19.2% recovery and 79.4% death. During the pandemic period (2020-2021), 123 episodes occurred in 106 people, with a predominance of primary bloodstream infection (43.1%) in females (50.9%), in the 17-39 age group (40.6%), those of mixed race (65.1%), and with hypertension (35.8%), with a 62.3% cure rate and a 37.7% death rate. There was persistently high overall carbapenem resistance in both periods. Between 2018 and 2019, *Klebsiella pneumoniae* (81.2%) and *Acinetobacter spp*. (78.6%) predominated. Between 2020 and 2021, resistance was found for *Klebsiella pneumoniae* (73.9%) and *Acinetobacter spp*. (80.0%).

**Conclusion:**

HAI in the ICU remained severe and associated with persistent antimicrobial resistance, mainly to multidrug-resistant *Klebsiella pneumoniae*. This emphasized the urgency of more effective prevention and control strategies in the hospital setting.

## Resumen

**Objetivo:**

Analizar las características demográficas y microbiológicas, así como los resultados clínicos de las personas con infecciones asociadas a la atención médica (IAAS) ingresadas en una unidad de cuidados intensivos (UCI) en el estado de Mato Grosso, Brasil, entre 2018 y 2021, comparando los períodos prepandemia y pandémico de COVID-19.

**Métodos:**

Estudio descriptivo y longitudinal con datos secundarios de personas diagnosticadas con IAAS. Se analizaron las IAAS, los microorganismos aislados, el perfil de sensibilidad, las comorbilidades, los resultados clínicos y los datos sociodemográficos.

**Resultados:**

Entre 2018 y 2021, 384 personas ingresaron en la UCI. Se registraron 218 episodios de IAAS en 179 de ellas. En el período prepandemia (2018-2019) se registraron 95 episodios en 73 personas, predominantemente asociados a: ventilación mecánica (48,4%), sexo masculino (65,8%), grupo etario 40-64 años (43,8%), mestizo (80,8%) e hipertenso (45,2%), con 19,2% de recuperación y 79,4% de fallecimiento. Durante el período pandémico (2020-2021), se presentaron 123 episodios en 106 personas, con predominio de bacteriemia (43,1%) en mujeres (50,9%), en el grupo de edad de 17 a 39 años (40,6%), en personas de raza mestiza (65,1%) y con hipertensión (35,8%), con una tasa de curación del 62,3% y una tasa de mortalidad del 37,7%. En ambos períodos se observó una resistencia general persistentemente alta a los carbapenémicos. Entre 2018 y 2019, predominaron *Klebsiella pneumoniae* (81,2%) y *Acinetobacter* spp. (78,6%). Entre 2020 y 2021, se observó resistencia en *Klebsiella pneumoniae* (73,9%) y *Acinetobacter* spp. (80,0%).

**Conclusión:**

Las IAAS en la UCI continuaron siendo graves y asociadas con resistencia antimicrobiana persistente, principalmente *Klebsiella pneumoniae* multirresistente. Esto reforzó la urgencia de implementar estrategias de prevención y control más eficaces en el ámbito hospitalario.

**Ethical aspects**

This research respected ethical principles, having obtained the following approval data::

Research ethics committee: Hospital Universitário Júlio Müller

Opinion number: 5,726,602

Approval date: 27/10/2022

Certificate of submission for ethical appraisal: 63186522.0.0000.5541

Informed consent form: Waived due to use of secondary data.

## Introduction 

Antimicrobial resistance is a process in which microorganisms adapt in response to exposure to antibiotic, antifungal and antiparasitic drugs (1). Indiscriminate use of these drugs intensifies selective pressure, which favors the emergence of multidrug-resistant strains, such as methicillin-resistant Staphylococcus aureus, carbapenem-resistant Klebsiella pneumoniae and Pseudomonas aeruginosa, mainly in intensive care units (ICU), making outbreak control difficult and increasing hospital morbidity and mortality.

Healthcare-associated infections (HAI) are a public health problem due to their increasing hospital costs, prolonging hospital stays and driving intensive use of antimicrobials. In ICU, the most frequently described HAI are ventilator-associated pneumonia, urinary tract infection, primary bloodstream infection and surgical site infection (2).

The World Health Organization (WHO) recommends rational use of antimicrobial drugs, involving appropriate therapy management, reduction of adverse events and patient safety, based on strategies such as Anatomical Therapeutic Chemical classes and defined daily dose of the drug in question (3).

In Brazil, a significant increase in antibiotic prescriptions was observed between 2014 and 2019, especially in the Midwest region, where 55% of prescriptions involved antimicrobials classified as inappropriate by the WHO Access, Watch and Reserve framework to combat antimicrobial resistance (4). The worrying growth in the inappropriate use of Watch drugs (those of higher resistance risk), such as ciprofloxacin, levofloxacin and combinations of amoxicillin with clavulanate, demonstrates the need for more rigorous surveillance policies and antimicrobial use management programs (4).

This scenario worsened during the COVID-19 pandemic, when empirical use of antibiotics was widely adopted, associated with hospital overload and prolonged hospital stays. This context contributed to the emergence of multidrug-resistant pathogens, such as methicillin-resistant Acinetobacter baumannii and Staphylococcus aureus (5). In 2015, an increase was recorded in HAI incidence and healthcare costs of up to 55% in critically ill infected individuals, compared to those without infection (6).

Specific methodological guidelines have been proposed to improve the quality of observational studies on antimicrobial resistance. The Strengthening the Reporting of Observational Studies in Epidemiology for Antimicrobial Stewardship protocol reinforces the importance of rigorously reporting the findings of observational studies in hospital settings, especially with regard to antimicrobial resistance. Its adoption enables greater standardization and quality in scientific communication, as well as supporting infection control and prevention actions in highly complex environments (7).

This study aimed to analyze the demographic and microbiological characteristics and clinical outcomes of people with HAIs admitted to an ICU in Mato Grosso state, Brazil, between 2018 and 2021, comparing the pre-COVID-19 and the COVID-19 pandemic periods.

## Methods 

### Design 

This is a descriptive study, based on clinical and laboratory data obtained from a review of medical records of people hospitalized in the ICU of a teaching hospital located in Mato Grosso state, from January 2018 to December 2021.

### Setting 

The study was conducted at the *Hospital Universitário Júlio* Müller in Cuiabá, the capital city of Mato Grosso state, an institution that, at the time, exclusively served users of the Brazilian Unified Health System (*Sistema Único de Saúde*, SUS). The hospital received people from various regions of the state and neighboring municipalities, with a high concentration of critical cases, especially in its Intensive Care Unit (ICU). It presented high incidence of HAI and multidrug-resistant microorganisms, which demanded rigorous surveillance and control measures. During the COVID-19 pandemic, the hospital was a state referral center for treatment of severe cases, having made 16 ICU beds available for this purpose.

### Population and eligibility criteria 

All individuals admitted to the ICU with diagnosis of HAI during the study period were included. There were no additional exclusion criteria other than incomplete records held on the case reporting systems or medical records.

### Data sources and variables 

The information was obtained from the case reporting forms of the Hospital Infection Control Service and the Brazilian Hospital Services Company (*Empresa Brasileira de Serviços Hospitalares*) hospital management application. Sociodemographic data (age, sex, schooling, race/skin color), clinical data (comorbidities, infection site, invasive procedures, antibiotics used, duration of pharmacological therapy, length of hospital stay), and laboratory data (blood cultures, urine cultures, tracheal secretion cultures, bronchoalveolar lavage and surgical wound secretions, microorganism identification, antibiograms, blood cultures) and data on the progression of the person’s health status (cure, death) were collected.

Microorganism identification and antibiotic susceptibility testing were performed using the automated VITEK 2 Compact system (bioMérieux, France), with interpretation according to the Brazilian Committee on Antimicrobial Susceptibility Testing (BrCAST). Blood cultures were processed using the BD Bactec FX40 system (Becton & Dickinson, United States). Results were classified as “sensitive”, “susceptible, increased exposure”, and “resistant”.

### Bias 

This study presented information bias inherent to the use of secondary records. Therefore, supplementary data were extracted from Hospital Infection Control Service documents. Data collection was carried out independently by two researchers in order to reduce record losses and inconsistencies.

### Outcomes 

The outcomes of interest were: characterization of HAI types; identification of etiological agents; and evaluation of the resistance profile of the isolated microorganisms. These outcomes were analyzed in association with demographic and clinical characteristics and with the COVID-19 pandemic period, confirmed by molecular biology examination reverse transcription-polymerase chain reaction or antigen test.

### Sample size

All cases reported between 2018 and 2021 were included. No prior sample size calculation was performed, as this was a study that analyzed secondary data.

### Statistical methods

The data were tabulated on Microsoft Excel spreadsheets and analyzed using EpiData Analysis v.2.2 software. In order to assess changes in the frequency of HAI related to the COVID-19 pandemic, individuals were stratified into two groups according to hospitalization period: pre-pandemic (2018-2019) and pandemic (2020-2021).

Absolute and relative HAI frequencies were described for each group. Differences between proportions were assessed using the chi-square test, taking a 5% significance level (α=0.05).

## Results 

Between 2018 and 2021, 384 people were hospitalized in the *Hospital Universitário Júlio* Müller ICU. In all, there were 218 episodes of HAI in 179 people (46.6%), 95 (43.6%) of which were in the pre-pandemic period (2018-2019), and 123 (56.4%) in the pandemic period (2020-2021).

In the two-year period prior to the pandemic, the predominant HAIs were ventilator-associated pneumonia (48.4%) and primary bloodstream infection (34.7%), observed in males (65.8%; p-value 0.014), people of mixed race/skin color (80,8%), people who were married (38,3%), aged 40-64 years (p-value 0.004) and ≥65 years (p-value<0.001). The main comorbidities found during this period were hypertension (45.2%) and diabetes (23.3%). Frequency of deaths among people with HAI was higher (79.4%; p-value<0.001) and there was a lower recorded cure rate (19.2%).

During the pandemic period, the predominant HAI were primary bloodstream infection (43.1%), ventilator-associated pneumonia (42.3%), and urinary tract infection (14.6%) in females (50.9%), those of Mixed race/skin color (65.1%), aged 17-39 years (40.6%), and in stable relationships (49.1%). The main comorbidities found in this period were hypertension (35.8%) and diabetes (21.7%), with lower frequency of death (37.7%) and higher frequency of cure (62.3%; p-value<0.001) (Table 1).

**Table 1:** Clinical and demographic data of individuals admitted to an adult intensive care unit with healthcare-associated infections during the pre-COVID-19 the COVID-19 pandemic periods. *Hospital Universitário Júlio Müller*, Mato Grosso, 2018-2021 (n=218)

Variables	2018-2019 n (%)	2020-2021 n (%)	p-value
Sex			0.014
Male	48 (65.8)	52 (49.1)	
Female	24 (32.8)	54 (50.9)	
No information	1 (1.4)	0	
**Age group** (years)			
17-39	12 (16.4)	43 (40.6)	-
40-64	32 (43.8)	38 (35.8)	0.004
≥65	28 (38.4)	25 (23.6)	<0.001
No information	1 (1.4)	0	
**Race/skin color**			
White	13 (17.8)	15 (14.2)	-
Mixed race	59 (80.8)	69 (65.1)	0.568
Asian	1 (1.4)	10 (9.4)	0.029
Indigenous	0	2 (1.9)	0.312
No information	0	10 (9.4)	
Schooling			
Incomplete elementary	31 (42.5)	22 (20.8)	-
Complete elementary	15 (20.5)	11 (10.4)	0.567
Complete high school	13 (17.8)	7 (6.6)	0.409
Higher education	1 (1.4)	5 (4.7)	0.063
No information	13 (17.8)	61 (57.5)	
**Marital status**			
Married	28 (38.3)	19 (17.9)	-
Single	25 (34.2)	35 (33.0)	0.049
Widowed	2 (2.7)	0	0.369
Stable union	13 (17.8)	52 (49.1)	<0.001
No information	5 (6.8)	0w	
Hypertension			0.135
Yes	33 (45.2)	38 (35.8)	
No	40 (54.8)	68 (64.2)	
Diabetes			0.470
Yes	17 (23.3)	23 (21.7)	
No	56 (76.7)	83 (78.3)	
**HIV**			0.186
Yes	4 (5.5)	2 (1.9)	
No	69 (95.5)	104 (98.1)	
**Chronic obstructive pulmonary disease**			0.447
Yes	6 (8.2)	7 (6.6)	
No	67 (91.8)	99 (93.4)	
**Autoimmune disease**			0.351
Yes	6 (8.2)	6 (5.7)	
No	67 (91.8)	100 (94.3)	
**Healthcare-related infections**	95	123	-
Ventilator-associated pneumonia	46 (48.4)	52 (42.3)	
Primary bloodstream infection	33 (34.7)	53 (43.1)	
Urinary tract infection	9 (9.5)	18 (14.6)	
Surgical site infection	6 (6.3)	0 (0.0)	
Infectious arthritis	1 (1.1)	0 (0.0)	
**Clinical outcome**	73	106	<0.001
Cure	14 (19.2)	66 (62.3)	
Death	58 (79.4)	40 (37.7)	
No information	1 (1.4)	0	

From 2018 to 2021, the predominant bacteria were Klebsiella pneumoniae (17.9%), coagulase-negative Staphylococcus (14.7%), and Pseudomonas aeruginosa (13.8%) (Table 2). During the same period, the most frequent microorganisms among those who died were Klebsiella pneumoniae (16.3%), coagulase-negative Staphylococcus (14.3%), Pseudomonas aeruginosa (13.3%), and Serratia marcescens (15.3%) (Table 3).

**Table 2:** Microorganisms causing healthcare-associated infections (HAI) in people admitted to an adult intensive care unit. *Hospital Universitário Júlio* Müller, Mato Grosso, 2018-2021 (n=218)

HAIs and specific agent	n (%)
*Klebsiella pneumoniae*	39 (17.9)
Coagulase-negative Staphylococcus^a^	32 (14.7)
*Pseudomonas spp*.	30 (13.8)
*Serratia marcescens*	21 (9.6)
*Acinetobacter spp*.	19 (8.7)
*Stenotrophomonas maltophilia*	13 (6.0)
*Burkholderia spp*.	15 (6.9)
*Enterococcus spp*.^b^	11 (5.0)
*Staphylococcus aureus*	4 (1.8)
Other Gram-negative bacteria^c^	7 (3.2)
*Streptococcus mitis*	1 (0.5)
*Candida albicans*	9 (4.1)
Non-*albicans Candida* ^d^	16 (7.3)
*Aspergillus spp*.	1 (0.5)

^a^Staphylococcus capitis=4; *Staphylococcus epidermidis*=21; *Staphylococcus haemolyticus*=3 and *Staphylococcus hominis*=4; ^b^Enterococcus faecalis=3; *Enterococcus faecium*=8; ^c^Elizabethkingea meningoseptica=4; *Morganella morganii*=1; *Sphingomonas paucimobilis*=1; *Aeromonas veronii*=1; ^d^Candida glabrata=4; *Candida guilliermondii*=2; *Candida krusei*=2; *Candida parapsilosis*=4 and *Candida tropicalis*=5.

**Table 3:** Microorganisms causing healthcare-associated infections (HAI) and death as the outcome in people admitted to an adult intensive care unit. *Hospital Universitário Júlio* Müller, Mato Grosso, 2018-2021 (n=218)

Microorganism causing HAI	Total (%)	Death due to HAI n (%)
Klebsiella pneumoniae	39 (17.9)	16 (16.3)
Coagulase-negative Staphylococcus^a^	32 (14.7)	14 (14.3)
*Pseudomonas spp*.	30 (13.8)	13 (13.3)
*Serratia marcescens*	21 (9.6)	15 (15.3)
*Acinetobacter spp*.	19 (8.7)	7 (7.1)
Non-*albicans Candida* ^b^	16 (7.3)	7 (7.1)
*Burkholderia spp*.	15 (6.9)	5 (5.1)
*Stenotrophomonas maltophilia*	13 (6.0)	9 (9.2)
*Enterococcus spp*.^c^	11 (5.0)	4 (4.1)
*Candida albicans*	9 (4.1)	3 (3.1)
*Staphylococcus aureus*	4 (1.8)	2 (2.0)
Other Gram-negative bacteria^d^	7 (3.2)	3 (3.1)
*Streptococcus mitis*	1 (0.5)	0
*Aspergillus spp*.	1 (0.5)	0
**Total**	218 (100)	98 (45.0)

^a^Coagulase-negative Staphylococcus: *Staphylococcus capitis*=4; *Staphylococcus epidermidis*=21; *Staphylococcus haemolyticus*=3 and *Staphylococcus hominis*=4. ^b^Candida glabrata=4; *Candida guilliermondii*=2; *Candida krusei*=2; *Candida parapsilosis*=4 and *Candida tropicalis*=5. ^c^Enterococcus faecalis=3 and *Enterococcus faecium*=8; ^d^Elizabethkingea meningoseptica=4; *Morganella morganii*=1 and *Sphingomonas paucimobilis*=1.

In the period prior to the pandemic, carbapenem-resistant strains of Klebsiella pneumoniae (81.2%), *Pseudomonas spp*. (69.2%), *Serratia marcescens* (100%) and *Acinetobacter spp*. (78.6%) predominated. During the pandemic, HAIs caused by strains of Klebsiella pneumoniae (73.9%), *Pseudomonas spp*. (58.8%), and *Acinetobacter spp*. (80.0%) remained carbapenem-resistant (Table 4).

**Table 4:** Resistance of microorganismos causing healthcare-associated infections in people admitted to an adult intensive care unit. *Hospital Universitário Júlio* Müller, Mato Grosso, 2018-2021

Variables	2018-2019 n (%)	2020-2021 n (%)
*Klebsiella pneumoniae*	16	23
Carbapenem-sensitive	2 (12.5)	6 (26.1)
Carbapenem-resistant	13 (81.2)	17 (73.9)
Not tested	1 (6.3)	0
Pseudomonas spp	13	17
Carbapenem-sensitive	4 (30.8)	7 (41.2)
Carbapenem-resistant	9 (69.2)	10 (58.8)
**Coagulase-negative** Staphylococcus		
Vancomycin-sensitive	11 (100.0)	21 (100.0)
Vancomycin-resistant	0	0
*Serratia marcescens*	19	2
Carbapenem-sensitive	0	2 (100.0)
Carbapenem-resistant	19 (100.0)	0
Acinetobacter spp	14	5
Carbapenem-sensitive	1 (7.1)	1 (20.0)
Carbapenem-resistant	11 (78.6)	4 (80.0)
Not tested	2 (14.3)	0
Non-*albicans Candida*	5	11
Fluconazole-sensitive	4 (80.0)	5 (45.4)
Fluconazole-resistant	1 (20.0)	2 (18.2)
Not tested	0	4 (36.4)
*Stenotrophomonas maltophilia^a^*	5	8
Sulfamethoxazole-sensitive	3 (60.0)	6 (75.0)
Sulfamethoxazole-resistant	1 (20.0)	2 (25.0)
Not tested	1 (20.0)	0
*Burkholderia* spp^b^	2	13
*Enterococcus spp*	2	9
Vancomycin-sensitive	2 (100.0)	5 (55.6)
Vancomycin-resistant	0	4 (44.4)
*Candida albicans*	2	7
Fluconazole-sensitive	2 (100.0)	7 (100.0)
Fluconazole-resistant	0	0
*Staphylococcus aureus*	3	1
Vancomycin-sensitive	3 (100.0)	1 (100.0)
Vancomycin-resistant	0	0
**Other Gram negative bacteria**	3	4
Carbapenem-sensitive	1 (33.3)	0
Carbapenem-resistant	2 (66.7)	3 (75.0)
Not tested	0	1 (25.0)
*Streptococcus mitis*		1
Vancomycin-sensitive	0	1 (100.0)
Vancomycin-resistant	0	0
Aspergillus spp^c^		1
Itraconazole-sensitive	0	0
Itraconazole-resistant	0	0

## Discussion 

This study identified an increase in HAI among young women during the COVID-19 pandemic. Throughout the period analyzed, there was a predominance of ventilator-associated pneumonia and primary bloodstream infection, both caused by carbapenem-resistant Klebsiella pneumoniae strains.

The highest number of deaths occurred in the period prior to the pandemic, possibly due to the hospitalization of more severely ill patients, the elderly, and those with uncertain prognosis. The higher recovery rate during the pandemic may be related to the fact that the *Hospital Universitário Júlio* Müller became a referral center in Mato Grosso for care in its ICU of pregnant women at high risk of COVID-19.

The limitations of this study relate to its retrospective nature, analysis of secondary data taken from medical records and infection control service reports, as well as inclusion of individuals with HAI in the ICU, which may generate information and selection biases.

In a hospital in the municipality of São Paulo, in the context of people hospitalized in its ICU, there was an increase in the prevalence of males with ventilator-associated pneumonia, urinary tract infection and primary bloodstream infection among the main HAIs observed among people who died during the COVID-19 pandemic period. These findings were associated with intensified use of invasive devices during the study period. A positive correlation was also observed between the prevalence of HAI and bed occupancy rates and inpatient days, which was attributed to the high healthcare demand imposed on the health system in the context of the pandemic in this region, corroborating the findings of this study (10-11).

In our analysis of HAI microbiological profile, predominance of carbapenem-resistant Klebsiella pneumoniae and Pseudomonas aeruginosa was observed throughout the investigation period of this study. This is consistent with findings that have identified these pathogens as important etiological agents that are difficult to manage clinically in ICU (12-14).

This study found a reduction in HAI cases caused by *Acinetobacter spp*., *Staphylococcus aureus* and *Serratia marcescens*, this being a phenomenon possibly related to the reconfiguration of the hospital as a COVID-19 referral unit in Mato Grosso. This may have optimized care flows, prevention protocols and, consequently, the altered spectrum of the microbiological profile observed.

These findings emphasized the importance of continuous monitoring of antimicrobial resistance and adaptation of HAI surveillance and control measures in health crisis scenarios.

In a hospital in Minas Gerais state, higher prevalence of *Acinetobacter baumannii*, *Staphylococcus aureus* and *Candida spp*. isolates was observed in people admitted to ICU beds designated for the treatment of COVID-19 during the first two years of the pandemic (14), which corroborated the findings of this study. However, during the pandemic, this study found incidence of HAIs caused by opportunistic microorganisms, which may have reflected changes in the profile of people treated at the teaching hospital Mato Grosso with greater clinical complexity associated with the critical care of people affected by COVID-19.

In the pre-pandemic period, there was an outbreak of HAIs caused by *Serratia marcescens* in the hospital studied in this article, which explains the higher frequency of this microorganism in this case series (16,17). In Paraná state, outbreaks of *Serratia marcescens* were identified in people with ventilator-associated pneumonia during the COVID-19 pandemic, demonstrating that the circulation of this pathogen can vary according to regional and institutional factors (18).

Use of central venous catheters, corticosteroid therapy and administering broad-spectrum antimicrobials have been described as important risk factors for increasing HAIs caused by invasive fungal infections. These represent a relevant complication in critically ill people with COVID-19, especially HAIs caused by non-*albicans Candida* and *Aspergillus spp*. (12).

In this study, in 2021, an increase in cases of HAIs caused by *Candida spp*. was observed during the pandemic period, in addition to an episode of ventilator-associated pneumonia caused by *Aspergillus spp*. These findings may be associated with the systemic use of corticosteroids and tocilizumab in the management of severe acute respiratory syndrome resulting from COVID-19 (9,12,14). Similarly, other opportunistic germs such as *Stenotrophomonas maltophilia* and *Burkholderia spp*. were also identified in this study, similar to what was identified in Lebanon with people hospitalized in ICU affected by severe COVID-19 (13).

These opportunistic microorganisms exhibit intrinsic resistance to several classes of antimicrobials due to selective pressure from the use of broad-spectrum antimicrobials in ICU, which limits available therapeutic options. Factors such as invasive mechanical ventilation, use of indwelling urinary catheters, insertion of central venous catheters and exposure to carbapenem and aminoglycoside class antibiotics are conditions that favor the occurrence of these infections. This increases the complexity of clinical management of HAI in critically ill people with worsening prognosis (19-20).

During the pandemic period, at the hospital in this study, there was an increase in infections caused by vancomycin-resistant *Enterococcus spp*., this being an HAI profile similar to that of ICU patients admitted to hospitals in Paraná state (18) and Salvador city (15,19). These multidrug-resistant strains have high dissemination capacity and contribute to the increasing occurrence of opportunistic infections. In the pre-pandemic period, all *Enterococcus spp*. strains were vancomycin-sensitive, while during the pandemic period, only half maintained this susceptibility, which stressed the relevance of continuous monitoring of antimicrobial resistance for management of ICU outbreaks and lower morbidity and mortality in critically ill patients (21-22).

This study highlighted the negative impact of the COVID-19 pandemic in terms of increased occurrence of HAI in the ICU. Multidrug-resistant *Klebsiella pneumoniae* stood out, with increased resistance to carbapenem antibiotics and vancomycin, as well as incidence of multidrug-resistant opportunistic microorganisms throughout the period analyzed. These findings emphasized the importance of epidemiological surveillance in elucidating cases and controlling HAI, in addition to stressing the urgency of more effective prevention and control strategies in the hospital environment.

## Data Availability

The data were obtained from medical records held on the Brazilian Hospital Services Company Teaching Hospitals Management Application, as well as physical medical records held at the *Hospital Universitário Júlio* Müller. Access to this data is available via the Mendeley Data: https://data.mendeley.com/datasets/vv93r9pkgf/1.
