# Supplementary material for: Healthcare-associated infections in an intensive care unit: a descriptive study, Mato Grosso state, Brazil, 2018-2021
Source: Epidemiol Serv Saude. 2026 Mar 30;35:e20250405. doi: 10.1590/S2237-96222026v35e20250405.en (PMC13037786; doi:10.1590/S2237-96222026v35e20250405.en)
Supplement: Translation

# Translation: Infecções relacionadas à assistência à saúde em unidade de terapia intensiva: estudo descritivo, Mato Grosso, 2018-2021

**DOI:** 10.1590/S2237-96222026v35e20250405.pt

**Authors:** Mayane Emanuelle Oliveira Fonseca, Francisco Kennedy Scofoni Faleiros Azevedo, Ana Luiza Rodrigues Silva, Marco Andrey Pepato, Wagner Izidoro de Brito, Eduarda Pavan, Cor Jesus Fernandes Fontes, Ruberlei Godinho de Oliveira

**Affiliations:** 1 Universidade Federal de Mato Grosso, Faculdade de Medicina, Cuiabá, MT, Brasil; 2 Hospital Universitário Júlio Müller, Mestrado Profissional em Ciências Aplicadas à Atenção Hospitalar, Cuiabá, MT, Brasil; 3 Secretaria de Estado de Saúde de Mato Grosso, Cuiabá, MT, Brasil

**Keywords:** Unidade de Terapia Intensiva, Serviços de Controle de Infecção Hospitalar, Resistência a Antimicrobianos, Covid-19, Estudos Retrospectivos

## Abstract

**Objetivo:**

Analisar as características demográficas e microbiológicas e os desfechos clínicos de pessoas com infecções relacionadas à assistência à saúde (IRAS) internadas em unidade de terapia intensiva (UTI) de Mato Grosso, entre 2018 e 2021, com a comparação dos períodos pré-pandemia de covid-19 e pandêmico.

**Métodos:**

Estudo descritivo e longitudinal com dados secundários de pessoas com diagnóstico de IRAS. Analisaram-se IRAS, microrganismos isolados, perfil de sensibilidade, comorbidades, desfechos clínicos e dados sociodemográficos.

**Resultados:**

Entre 2018 e 2021, 384 pessoas foram internadas em UTI. Houve 218 episódios de IRAS em 179 delas. Na pré-pandemia (2018-2019), registraram-se 95 episódios em 73 pessoas, com predomínio de pneumonia associada a: ventilação mecânica (48,4%), sexo masculino (65,8%), faixa etária 40-64 anos (43,8%), pardos (80,8%) e hipertensos (45,2%), com 19,2% de cura e 79,4% de óbito. No período pandêmico (2020-2021), ocorreram 123 episódios em 106 pessoas, com predomínio da infecção primária de corrente sanguínea (43,1%) em pessoas do sexo feminino (50,9%), na faixa etária 17-39 anos (40,6%), pardas (65,1%) e hipertensas (35,8%), com 62,3% de cura e 37,7% de óbito. Houve resistência global a carbapenêmicos persistentemente elevada em ambos os períodos. Entre 2018 e 2019, predominaram a *Klebsiella pneumoniae* (81,2%) e a Acinetobacter spp. (78,6%). Entre 2020 e 2021, houve resistência em *Klebsiella pneumoniae* (73,9%) e em Acinetobacter spp. (80,0%).

**Conclusão:**

As IRAS em UTI permaneceram graves e associadas à resistência microbiana persistente, principalmente a *Klebsiella pneumoniae* multirresistente. Isso reforçou a urgência por estratégias de prevenção e de controle mais efetivo no ambiente hospitalar.

**Aspectos éticos**

Esta pesquisa respeitou os princípios éticos, obtendo os seguintes dados de aprovação: :

Comitê de ética em pesquisa: Hospital Universitário Júlio Müller

Número do parecer: 5.726.602

Data de aprovação: 27/10/2022

Certificado de apresentação de apreciação ética: 63186522.0.0000.5541

Registro do consentimento livre e esclarecido: Dispensado devido à coleta de dados secundários.

## Introdução 

A resistência microbiana é um processo adaptativo dos microrganismos à exposição a antibióticos, antifúngicos e antiparasitários (1). O uso indiscriminado desses fármacos intensifica a pressão seletiva, o que favorece o surgimento de cepas multirresistentes, como *Staphylococcus aureus*, resistente à meticilina, *Klebsiella pneumoniae* e *Pseudomonas aeruginosa* resistentes aos carbapenêmicos, principalmente em unidades de terapia intensiva (UTI), o que dificulta o controle de surtos e aumenta a morbimortalidade hospitalar. 

As infecções relacionadas à assistência à saúde (IRAS) representam um problema de saúde pública por elevarem os custos hospitalares, prolongarem o tempo de internação e por impulsionarem o uso intensivo de antimicrobianos. Em UTI, as IRAS descritas com mais frequência são a pneumonia associada à ventilação mecânica, a infecção do trato urinário, a infecção primária de corrente sanguínea e a infecção de sítio cirúrgico (2). 

A Organização Mundial da Saúde (OMS) recomenda o uso racional de antimicrobianos através do gerenciamento adequado da terapêutica, da redução de eventos adversos e da segurança da pessoa, com base em estratégias como a classificação anatômica terapêutica química e a dose diária definida do fármaco (3).

No Brasil, observou-se aumento significativo na prescrição de antibióticos entre 2014 e 2019, especialmente na região Centro-Oeste, onde 55% das prescrições envolviam antimicrobianos classificados como inadequados pelo método de Acesso, Vigilância e Reserva proposto pela OMS para monitorar o uso de antibióticos quanto ao acesso, à vigilância e à exceção (4). O crescimento preocupante no uso inadequado de fármacos em vigilância, como ciprofloxacino, levofloxacino e combinações de amoxicilina com clavulanato, demonstra a necessidade de políticas mais rigorosas de vigilância e de programas de gerenciamento do uso de antimicrobianos (4). 

Esse cenário se agravou durante a pandemia de covid-19, quando o uso empírico de antibióticos foi amplamente adotado, associado à sobrecarga hospitalar e ao tempo prolongado de internações. Tal contexto contribuiu para a emergência de patógenos multirresistentes, como *Acinetobacter baumannii* e *Staphylococcus aureus*, resistentes à meticilina (5). Em 2015, houve registro de aumento de incidência das IRAS e dos custos assistenciais em até 55% em pessoas em estado crítico infectadas, quando comparadas àquelas sem infecção (6).

Diretrizes metodológicas específicas têm sido propostas para aprimorar a qualidade dos estudos observacionais sobre resistência antimicrobiana. O protocolo Strengthening the Reporting of Observational Studies in Epidemiology for Antimicrobial Stewardship reforça a importância de relatar com rigor os achados de estudos observacionais em ambientes hospitalares, especialmente no que se diz respeito à resistência antimicrobiana. Sua adoção possibilita maior padronização e qualidade na comunicação científica, além de subsidiar ações de controle e prevenção de infecções em ambientes de alta complexidade (7).

Este estudo teve como objetivo analisar as características demográficas e as microbiológicas e os desfechos clínicos de pessoas com IRAS internadas em UTI de Mato Grosso, entre 2018 e 2021, com a comparação dos períodos pré-pandemia de covid-19 e pandêmico.

## Métodos 

### Delineamento 

Trata-se de estudo descritivo, baseado em dados clínicos e laboratoriais obtidos a partir da revisão de prontuários de pessoas hospitalizadas em UTI de um hospital universitário localizado em Mato Grosso, no período de janeiro de 2018 a dezembro de 2021.

### Local e contexto

O estudo foi realizado no Hospital Universitário Júlio Müller, em Cuiabá, Mato Grosso, instituição que, à época, atendia exclusivamente usuários do Sistema Único de Saúde. O hospital recebia pessoas das diversas regiões do estado e de municípios vizinhos, com a concentração de grande demanda de casos críticos, especialmente em UTI. Ele apresentava elevada incidência de IRAS e de microrganismos multirresistentes, o que demandava medidas rigorosas de vigilância e de controle. Durante a pandemia de covid-19, o hospital foi referência estadual para o tratamento de casos graves, tendo disponibilizado 16 leitos de UTI para esse fim.

### População e critérios de elegibilidade

Foram incluídas todas as pessoas admitidas na UTI com diagnóstico de IRAS no período estudado. Não houve critérios de exclusão adicionais além de registros incompletos nos sistemas de notificação ou nos prontuários médicos.

### Fontes de dados e variáveis

As informações foram obtidas a partir das fichas de notificação do Serviço de Controle de Infecção Hospitalar do hospital e do sistema de aplicativo de gestão da Empresa Brasileira de Serviços Hospitalares. Foram coletados os dados sociodemográficos (idade, sexo, escolaridade, raça/cor da pele), clínicos (comorbidades, sítio de infecção, procedimentos invasivos, antibióticos utilizados, tempo de uso da terapia farmacológica, tempo de internação) e laboratoriais (culturas de sangue, urina, secreção traqueal, lavado broncoalveolar e secreções de ferida operatória, identificação de microrganismos, antibiogramas, hemoculturas) e os dados sobre a evolução do quadro de saúde da pessoa (cura, óbito).

A identificação dos microrganismos e o teste de suscetibilidade aos antibióticos foram realizados pelo sistema automatizado VITEK 2 Compact (bioMérieux, França), com interpretação segundo o Brazilian Committee on Antimicrobial Susceptibility Testing (BrCAST). As hemoculturas foram processadas pelo sistema BD Bactec FX40 (Becton & Dickinson, Estados Unidos). Os resultados foram classificados como “sensível”, “sensível com necessidade de aumento da exposição” e “resistente”.

### Viés 

Este estudo apresentou viés de informação inerente ao uso de registros secundários. Por isso, dados complementares foram extraídos de documentos do Serviço de Controle de Infecção Hospitalar. A coleta foi realizada de forma independente por dois pesquisadores, a fim de reduzir perdas e inconsistências nos registros.

### Desfechos 

Os desfechos de interesse foram: caracterização dos tipos de IRAS; identificação dos agentes etiológicos; e avaliação do perfil de resistência dos microrganismos isolados. Esses desfechos foram analisados em associação com características demográficas e clínicas e com o período pandêmico de covid-19, confirmado por exame de biologia molecular reação em cadeia da polimerase com transcrição reversa ou teste de antígeno.

### Tamanho amostral

Foram incluídos todos os casos notificados no período 2018-2021. Não foi realizado cálculo amostral prévio, por se tratar de análise de base de dados secundários.

### Métodos estatísticos

Os dados foram tabulados em planilhas do Microsoft Excel e analisados no software EpiData Analysis v.2.2. Para avaliar alterações na frequência de IRAS relacionadas à pandemia de covid-19, as pessoas foram estratificadas em dois grupos conforme o período de hospitalização: pré-pandemia (2018-2019) e pandêmico (2020-2021). 

Foram descritas frequências absolutas e relativas de IRAS para cada grupo. Diferenças entre proporções foram avaliadas pelo teste do qui-quadrado, com nível de significância de 5% (α=0,05).

## Resultados 

Entre 2018 e 2021, 384 pessoas foram hospitalizadas na UTI do Hospital Universitário Júlio Müller. No total, foram 218 episódios de IRAS em 179 pessoas (46,6%), das quais 95 (43,6%) foram no biênio pré-pandemia (2018-2019), e 123 (56,4%), no biênio pandêmico (2020-2021). 

No biênio pré-pandêmico, as IRAS predominantes foram pneumonia associada à ventilação mecânica (48,4%) e infecção primária de corrente sanguínea (34,7%), percebidas em pessoas do sexo masculino (65,8%; p-valor 0,014), de raça/cor da pele parda (80,8%), casados (38,3%), de faixa etária 40-64 anos (p-valor 0,004) e ≥65 anos (p-valor<0,001). As principais comorbidades das pessoas desse período foram hipertensão (45,2%) e diabetes (23,3%). A frequência de óbitos de pessoas com IRAS foi maior (79,4%; p-valor<0,001) e houve menor registro de cura (19,2%).

No biênio pandêmico, as IRAS predominantes foram infecção primária de corrente sanguínea (43,1%), pneumonia associada à ventilação mecânica (42,3%) e infecção do trato urinário (14,6%) em pessoas do sexo feminino (50,9%), de raça/cor da pele parda (65,1%), com faixa etária 17-39 anos (40,6%) e em união estável (49,1%). As principais comorbidades das pessoas desse período foram hipertensão (35,8%) e diabetes (21,7%), com registro de menor frequência de óbito (37,7%) e de maior frequência de cura (62,3%; p-valor<0,001) (Tabela 1).

**Tabela 1:** Dados clínicos e demográficos das pessoas internadas em unidade de terapia intensiva adulta com infecções relacionadas à assistência à saúde nos períodos pré-pandemia de covid-19 e pandêmico. Hospital Universitário Júlio Müller, Mato Grosso, 2018-2021 (n=218)

Variáveis	2018-2019 n (%)	2020-2021 n (%)	p-valor
Sexo			0,014
Masculino	48 (65,8)	52 (49,1)	
Feminino	24 (32,8)	54 (50,9)	
Sem informações	1 (1,4)	0	
**Faixa etária** (anos)			
17-39	12 (16,4)	43 (40,6)	-
40-64	32 (43,8)	38 (35,8)	0,004
≥65	28 (38,4)	25 (23,6)	<0,001
Sem informações	1 (1,4)	0	
**Raça/cor da pele**			
Branca	13 (17,8)	15 (14,2)	-
Parda	59 (80,8)	69 (65,1)	0,568
Amarela	1 (1,4)	10 (9,4)	0,029
Indígena	0	2 (1,9)	0,312
Sem informações	0	10 (9,4)	
Escolaridade			
1º grau incompleto	31 (42,5)	22 (20,8)	-
1º grau completo	15 (20,5)	11 (10,4)	0,567
2º grau completo	13 (17,8)	7 (6,6)	0,409
Superior	1 (1,4)	5 (4,7)	0,063
Sem informações	13 (17,8)	61 (57,5)	
**Estado civil**			
Casado	28 (38,3)	19 (17,9)	-
Solteiro	25 (34,2)	35 (33,0)	0,049
Viúvo	2 (2,7)	0	0,369
União estável	13 (17,8)	52 (49,1)	<0,001
Sem informações	5 (6,8)	0	
Hipertensão			0,135
Sim	33 (45,2)	38 (35,8)	
Não	40 (54,8)	68 (64,2)	
Diabetes			0,470
Sim	17 (23,3)	23 (21,7)	
Não	56 (76,7)	83 (78,3)	
HIV			0,186
Sim	4 (5,5)	2 (1,9)	
Não	69 (95,5)	104 (98,1)	
**Doença pulmonar obstrutiva crônica**			0,447
Sim	6 (8,2)	7 (6,6)	
Não	67 (91,8)	99 (93,4)	
**Doença autoimune**			0,351
Sim	6 (8,2)	6 (5,7)	
Não	67 (91,8)	100 (94,3)	
**Infecções relacionadas à assistência à saúde**	95	123	-
**Pneumonia associada à ventilação mecânica**	46 (48,4)	52 (42,3)	
**Infecção primária de corrente sanguínea**	33 (34,7)	53 (43,1)	
**Infecção do trato urinário**	9 (9,5)	18 (14,6)	
**Infecção de sítio cirúrgico**	6 (6,3)	0 (0,0)	
**Pioartrite**	1 (1,1)	0 (0,0)	
**Desfecho clínico**	73	106	<0,001
Cura	14 (19,2)	66 (62,3)	
Óbito	58 (79,4)	40 (37,7)	
Sem informações	1 (1,4)	0	

De 2018 a 2021, houve predomínio das bactérias *Klebsiella pneumoniae* (17,9%), Staphylococcus coagulase negativo (14,7%) e *Pseudomonas aeruginosa* (13,8%) (Tabela 2). Nesse mesmo período, os microrganismos mais frequentes entre as pessoas que evoluíram para óbito foram *Klebsiella pneumoniae* (16,3%), Staphylococcus coagulase negativo (14,3%), *Pseudomonas aeruginosa* (13,3%) e *Serratia marcescens* (15,3%) (Tabela 3).

**Tabela 2:** Microrganismos causadores de infecções relacionadas à assistência à saúde (IRAS) nas pessoas internadas em unidade de terapia intensiva adulta. Hospital Universitário Júlio Müller, Mato Grosso, 2018-2021 (n=218)

IRAS e o agente específico	n (%)
*Klebsiella pneumoniae*	39 (17,9)
*Staphylococcus coagulase ^n^egativoa*	32 (14,7)
*Pseudomonas spp*.	30 (13,8)
*Serratia marcescens*	21 (9,6)
*Acinetobacter spp*.	19 (8,7)
*Stenotrophomonas maltophilia*	13 (6,0)
*Burkholderia spp*.	15 (6,9)
*Enterococcus spp*.^b^	11 (5,0)
*Staphylococcus aureus*	4 (1,8)
Outras bactérias Gram negativas^c^	7 (3,2)
*Streptococcus mitis*	1 (0,5)
*Candida albicans*	9 (4,1)
*Candida não albicans* ^d^	16 (7,3)
*Aspergillus spp*.	1 (0,5)

**Tabela 3:** Microrganismos causadores de infecções relacionadas à assistência à saúde (IRAS) e do desfecho óbito nas pessoas internadas em unidade de terapia intensiva adulta. Hospital Universitário Júlio Müller, Mato Grosso, 2018-2021 (n=218)

Microrganismo causador de IRAS	Total (%)	Óbito de pessoas com IRAS n (%)
*Klebsiella pneumoniae*	39 (17,9)	16 (16,3)
Staphylococcus coagulase negativo (SCN)^a^	32 (14,7)	14 (14,3)
*Pseudomonas spp*.	30 (13,8)	13 (13,3)
*Serratia marcescens*	21 (9,6)	15 (15,3)
*Acinetobacter spp*.	19 (8,7)	7 (7,1)
*Candida* não albicans^b^	16 (7,3)	7 (7,1)
*Burkholderia spp*.	15 (6,9)	5 (5,1)
*Stenotrophomonas maltophilia*	13 (6,0)	9 (9,2)
*Enterococcus spp*.^c^	11 (5,0)	4 (4,1)
*Candida albicans*	9 (4,1)	3 (3,1)
*Staphylococcus aureus*	4 (1,8)	2 (2,0)
Outras bactérias Gram negativas^d^	7 (3,2)	3 (3,1)
*Streptococcus mitis*	1 (0,5)	0
*Aspergillus spp*.	1 (0,5)	0
Total	218 (100)	98 (45,0)

No período pré-pandemia, foram predominantes cepas de *Klebsiella pneumoniae* (81,2%), *Pseudomonas spp*. (69,2%), *Serratia marcescens* (100%) e *Acinetobacter spp*. (78,6%) resistentes aos carbapenêmicos. No período pandêmico, mantiveram-se resistentes aos carbapenêmicos as IRAS ocasionadas por cepas de *Klebsiella pneumoniae* (73,9%), *Pseudomonas spp*. (58,8%) e *Acinetobacter spp*. (80,0%) (Tabela 4).

**Tabela 4:** Resistência dos microrganismos causadores de infecções relacionadas à assistência à saúde nas pessoas internadas em unidade de terapia intensiva adulta. Hospital Universitário Júlio Müller, Mato Grosso, 2018-2021

Variáveis	2018-2019 n (%)	2020-2021 n (%)
*Klebsiella pneumoniae*	16	23
Sensível a carbapenêmicos	2 (12,5)	6 (26,1)
Resistente a carbapenêmicos	13 (81,2)	17 (73,9)
Não testado	1 (6,3)	0
*Pseudomonas* spp.	13	17
Sensível a carbapenêmicos	4 (30,8)	7 (41,2)
Resistente a carbapenêmicos	9 (69,2)	10 (58,8)
Staphylococcus **coagulase negativo**		
Sensível vancomicina	11 (100,0)	21 (100,0)
Resistente vancomicina	0	0
*Serratia marcescens*	19	2
Sensível a carbapenêmicos	0	2 (100,0)
Resistente a carbapenêmicos	19 (100,0)	0
*Acinetobacter* spp.	14	5
Sensível a carbapenêmicos	1 (7,1)	1 (20,0)
Resistente a carbapenêmicos	11 (78,6)	4 (80,0)
Não testado	2 (14,3)	0
Candida não albicans	5	11
Sensível ao fluconazol	4 (80,0)	5 (45,4)
Resistente ao fluconazol	1 (20,0)	2 (18,2)
Não testado	0	4 (36,4)
*Stenotrophomonas maltophilia^a^*	5	8
Sensível a sulfametoxazol	3 (60,0)	6 (75,0)
Resistente a sulfametoxazol	1 (20,0)	2 (25,0)
Não testado	1 (20,0)	0
*Burkholderia* spp.^b^	2	13
*Enterococcus spp*	2	9
Sensível à vancomicina	2 (100,0)	5 (55,6)
Resistente à vancomicina	0	4 (44,4)
*Candida albicans*	2	7
Sensível ao fluconazol	2 (100,0)	7 (100,0)
Resistente ao fluconazol	0	0
*Staphylococcus aureus*	3	1
Sensível à vancomicina	3 (100,0)	1 (100,0)
Resistente à vancomicina	0	0
**Outras bactérias Gram negativas**	3	4
Sensível a carbapenêmicos	1 (33,3)	0
Resistente a carbapenêmicos	2 (66,7)	3 (75,0)
Não testado	0	1 (25,0)
*Streptococcus mitis*		1
Sensível à vancomicina	0	1 (100,0)
Resistente à vancomicina	0	0
*Aspergillus* spp.^c^		1
Sensível ao itraconazol	0	0
Resistente ao itraconazol	0	0

^a^Levofloxacina e sulfametoxal – trimetropim não foram testados; ^b^Levofloxacina e carbapenêmicos não foram testados; ^c^Não foi realizado antifungigrama.

## Discussão 

Este estudo identificou aumento de IRAS em mulheres jovens durante a pandemia de covid-19. Em todo o período analisado, houve predomínio de pneumonia associada à ventilação mecânica e de infecção primária da corrente sanguínea, ambas causadas por cepas de *Klebsiella pneumoniae* resistentes a carbapenêmicos. 

O maior número de óbitos ocorreu no período pré-pandêmico, possivelmente devido à internação de pessoas mais graves, de idosos e com prognóstico reservado. A maior frequência de cura no período pandêmico pode estar relacionada ao fato de o Hospital Universitário Júlio Müller ter se tornado referência em Mato Grosso para atendimento de gestantes de alto risco na UTI para covid-19.

As limitações deste estudo se apresentaram no seu caráter retrospectivo, na análise de dados secundários de prontuários e de relatórios do serviço de controle de infecção e na inclusão de pessoas com IRAS em UTI, o que pode gerar vieses de informação e de seleção.

Em um hospital do município de São Paulo, no contexto das pessoas internadas em UTI, houve aumento da prevalência de pessoas do sexo masculino com pneumonia associada à ventilação mecânica, infecção do trato urinário e infecção primária da corrente sanguínea entre as principais IRAS observadas entre as pessoas que evoluíram para óbito durante o período pandêmico. Esses achados foram associados ao uso intensificado de dispositivos invasivos no período deste estudo. Também foi observada correlação positiva entre a prevalência de IRAS e os índices de ocupação de leitos e de pessoas-dia, o que foi atribuído à elevada demanda assistencial imposta ao sistema de saúde no contexto da pandemia nessa região, corroborando os achados deste estudo (10-11).

Na análise do perfil microbiológico das IRAS, observou-se predomínio de *Klebsiella pneumoniae* e de *Pseudomonas aeruginosa* resistentes aos carbapenêmicos em todo o período de investigação deste estudo. Isso está em consonância com achados que apontaram tais patógenos como importantes agentes etiológicos de difícil manejo clínico em UTI (12-14). 

Neste estudo, houve redução dos casos de IRAS por Acinetobacter spp., *Staphylococcus aureus* e *Serratia marcescens*, fenômeno possivelmente relacionado à reconfiguração do hospital como unidade de referência para covid-19 em Mato Grosso. Isso pode ter otimizados os fluxos assistenciais, os protocolos de prevenção e, consequentemente, o espectro alterado do perfil microbiológico observado. 

Estes achados reforçaram a importância do monitoramento contínuo da resistência antimicrobiana e da adaptação das medidas de cultura de vigilância e de controle de IRAS em cenários de crise sanitária.

Em um hospital de Minas Gerais, foi evidenciada maior prevalência de isolados de *Acinetobacter baumannii*, *Staphylococcus aureus* e Candida spp. em pessoas internadas em leitos de UTI destinadas ao tratamento da covid-19 durante o primeiro biênio pandêmico (14), o que corroborou os achados deste estudo. Contudo, durante a pandemia, este estudo verificou incidência de IRAS ocasionadas por microrganismos oportunistas, o que pode ter refletido as mudanças no perfil das pessoas atendidas no hospital universitário de Mato Grosso com maior complexidade clínica associada ao cuidado crítico das pessoas acometidas pela covid-19.

No período pré-pandemia, houve um surto de IRAS por *Serratia marcescens* no hospital deste estudo, o que justificou a maior frequência desse microrganismo nesta casuística (16,17). No Paraná, foram identificados surtos de *Serratia marcescens* em pessoas com pneumonia associada à ventilação mecânica durante a pandemia de covid-19, o que demonstrou que a circulação desse patógeno pode variar de acordo com fatores regionais e institucionais (18).

O uso de cateteres venosos centrais, a corticoterapia e a administração de antimicrobianos de amplo espectro têm sido descritos como fatores de risco importantes no aumento de IRAS ocasionadas por infecções fúngicas invasivas. Estas representam uma complicação relevante em pessoas em estado crítico com covid-19, especialmente as IRAS ocasionadas por Candida não albicans e por *Aspergillus spp*. (12). 

Neste estudo, em 2021, observou-se aumento de casos de IRAS por *Candida spp*. no período pandêmico, além de um episódio de pneumonia associada à ventilação mecânica causada por *Aspergillus spp*. Esses achados podem estar associados ao uso sistêmico de corticoides e tocilizumabe no manejo da síndrome respiratória aguda grave decorrente da covid-19 (9,12,14). De forma semelhante, outros germes oportunistas como *Stenotrophomonas maltophilia* e *Burkholderia spp*. também foram identificados neste estudo, semelhante à identificação feita no Líbano com pessoas internadas em UTI acometidas pela covid-19 grave (13). 

Esses microrganismos oportunistas apresentam resistência intrínseca a diversas classes de antimicrobianos devido à pressão seletiva pelo uso de antimicrobianos de amplo espectro na UTI, o que limita as opções terapêuticas disponíveis. Fatores como ventilação mecânica invasiva, uso de cateter vesical de demora, inserção de cateter venoso central e exposição aos antibióticos das classes dos carbapenêmicos e dos aminoglicosídeos configuram condições que favorecem a ocorrência dessas infecções. Isso aumenta a complexidade do manejo clínico das IRAS nas pessoas em estado crítico com piora prognóstica (19-20).

No período pandêmico, no hospital deste estudo, houve aumento das infecções por Enterococcus spp. resistentes à vancomicina; um perfil de IRAS semelhante ao de pessoas de UTI internadas em hospital do Paraná (18) e de Salvador (15,19). Essas cepas multirresistentes apresentam alta capacidade de disseminação e contribuem para a ocorrência crescente de infecções oportunistas. No período pré-pandemia, todas as cepas de Enterococcus spp. foram sensíveis à vancomicina, enquanto, no período pandêmico, apenas metade manteve essa suscetibilidade, o que reforçou a relevância do monitoramento contínuo da resistência antimicrobiana para o manejo de surtos em UTI e a menor morbimortalidade nas pessoas em estado crítico (21-22).

Este estudo evidenciou o impacto negativo da pandemia de covid-19 através do aumento da ocorrência de IRAS em UTI. Destacou-se *Klebsiella pneumoniae* multirresistente com o aumento da resistência aos antibióticos carbapenêmicos e à vancomicina, bem como incidência de microrganismos oportunistas multirresistentes em todo o período analisado. Tais achados reforçaram a importância da vigilância epidemiológica na elucidação dos casos e no controle das IRAS, além de reforçar a urgência de estratégias de prevenção e de controle mais efetivas no ambiente hospitalar.

## Data Availability

Os dados foram obtidos a partir de prontuários disponíveis no Aplicativo de Gestão para Hospitais Universitários, da Empresa Brasileira de Serviços Hospitalares, além de prontuários físicos do Hospital Universitário Júlio Müller. O acesso a esses dados está disponível no repositório Mendeley Data: https://data.mendeley.com/datasets/vv93r9pkgf/1.
